# Comparative study of resistance to antibiotics of strains of *Klebsiella* isolated in urinary and respiratory infections

**DOI:** 10.1186/1471-2334-13-S1-P39

**Published:** 2013-12-16

**Authors:** Dan Dumitru Hurezeanu, Livia Dragonu, Carmen Canciovici, Daniela Ristea, Doina Ene, Mioara Cotulbea, Magdalena Peter, Felicia Dumitraşcu, Maria Bălan

**Affiliations:** 1 University of Medicine and Pharmacy Craiova, Romania; 2 “Victor Babeş” Clinical Hospital of Infectious Diseases and Pneumology, Craiova, Romania; 3 Emergency Clinical Hospital, Craiova, Romania

## Background

A study of the dynamics of antibiotic resistance of *Klebsiella pneumoniae* is necessary in order to adapt therapeutic recommendations.

The objectives of this paper pursue the comparative analysis of resistance to antibiotics of strains of *Klebsiella pneumoniae* isolated in urinary and respiratory tract infections.

## Methods

Retrospective study conducted between 01 January 2013 – 31 August 2013 on the resistance of *Klebsiella pneumoniae* isolated from sputum (96 strains) and urine culture (163 strains) from patients hospitalized in the “Victor Babeş” Clinical Hospital of Infectious Diseases and Pneumology, Craiova. The antibiogram was performed by classical diffusimetric method.

## Results

The resistance to antibiotics was higher in *Klebsiella pneumoniae* strains isolated from urine culture compared to those isolated in respiratory infections in case of: a) beta-lactams (ceftriaxone 45.2% versus 69.5%, cefaclor 36.7% versus 52.9%, cefuroxime 49.3% versus 61.9%, cefoperazone-sulbactam 70.6% versus 85.7%, piperacillin-tazobactam 58.1% versus 75.5%, aztreonam 45.8% versus 66.6%, ampicillin-sulbactam 46.4% versus 69.1%, amoxicillin-clavulanic acid 26.8% versus 40.5%), b) quinolones (ciprofloxacin 61.8% versus 83.4%), c) co-trimoxazole (45.4% versus 82.8%). The sensitivity profile showed no major differences for: colistin (82.7% versus 81.6%), carbapenems (95.2% versus 97.8%) and aminoglycosides (72.3% versus 75.8%).

## Conclusion

The sensitivity of *Klebsiella pneumoniae* to carbapenems remained high, recommending the use of these antibiotics for infections with resistant germs. The empirical use of antibiotics for urinary tract infections and invasive urological maneuvers may explain the increased resistance profile of germs isolated from urine culture.

